# Establishing a Field-Effect Transistor Sensor for the Detection of Mutations in the Tumour Protein 53 Gene (TP53)—An Electrochemical Optimisation Approach

**DOI:** 10.3390/bios9040141

**Published:** 2019-12-06

**Authors:** Lisa Crossley, Bukola Attoye, Vincent Vezza, Ewen Blair, Damion K. Corrigan, Stuart Hannah

**Affiliations:** Department of Biomedical Engineering, University of Strathclyde, 40 George Street, Glasgow G1 1QE, UK; lisa.crossley.2015@uni.strath.ac.uk (L.C.); bukola.omolaiye@strath.ac.uk (B.A.); vincent.vezza@strath.ac.uk (V.V.); ewen.blair@strath.ac.uk (E.B.); damion.corrigan@strath.ac.uk (D.K.C.)

**Keywords:** field-effect transistor, biosensor, TP53, electrochemistry, open-circuit potential

## Abstract

We present a low-cost, sensitive and specific DNA field-effect transistor sensor for the rapid detection of a common mutation to the tumour protein 53 gene (TP53). The sensor consists of a commercially available, low-cost, field-effect transistor attached in series to a gold electrode sensing pad for DNA hybridisation. The sensor has been predominantly optimised electrochemically, particularly with respect to open-circuit potentiometry as a route towards understanding potential (voltage) changes upon DNA hybridisation using a transistor. The developed sensor responds sensitively to TP53 mutant DNA as low as 100 nM concentration. The sensor responds linearly as a function of DNA target concentration and is able to differentiate between complementary and noncomplementary DNA target sequences.

## 1. Introduction

Circulating tumour DNA (ctDNA) is an emerging biomarker for cancer which has the potential for early detection and monitoring of treatment response [1]. Rapid and accurate detection of important mutations, for example, tumour protein 53 (TP53), from the ctDNA fraction in a point-of-care (PoC) manner represents a significant analytical challenge. To be able to detect a relatively small fraction of blood-borne DNA fragments originating from a tumour and carrying cancerous mutations against the background of the total circulating DNA requires the production of a device with both high sensitivity and specificity [2]. The current gold standard for tumour diagnosis is tissue biopsy; however, there is a clear need for a more effective method as it does not reliably detect early stage tumours or lesions [3]. The efficacy of tissue biopsy for monitoring changes to tumours throughout treatment is limited as the tissue sample reflects its molecular composition at the point of retrieval which means that by the time results are obtained, the tumour will have likely changed genetically. In addition, only one area of a tumour is characterised from a tissue biopsy, which is not ideal as different areas of a tumour can have different genetic profiles [4]. Therefore, a liquid-based approach to biopsy based on ctDNA could be revolutionary in early cancer diagnosis as living tumour cells are the final mechanism by which ctDNA can enter the blood [3]. Based on ctDNA analysis, real-time monitoring of tumours could be achieved. Therefore, it was decided to begin development of such a sensor using the genetic sequence from TP53 mutation, with which many types of cancer are associated. This mutation is useful to study because it correlates very closely with disease severity and is commonly employed in multiplex assays of ctDNA.

In recent years, there has been a growing demand for rapid PoC molecular diagnostic tests for a wide variety of diagnostic applications, including DNA detection [5,6,7]. The aim of such PoC tests is for them to be performed outside of the laboratory, either at the patient’s bedside or near-bedside, and could be of particular advantage in low/middle-income countries where resources/technical knowledge are limited, and early detection of various infectious diseases is critical to human health [8,9,10]. Useful PoC tests must be low-cost, portable, simple to use, sensitive and specific to the particular biomarker of interest.

To date, many biosensors exist which feature different detection methods including optical techniques such as optical fibres to detect DNA or bacteria [11,12]. However, the drawbacks of optically based techniques are their cost-effectiveness, complexity and relative difficulty to implement into a PoC test. Electrochemical-based sensors, on the other hand, are a promising technology for use at the point of care and offer label-free detection, low-cost production, integration capabilities with technology such as microfluidics and can be integrated with low-cost electronics to provide real-time data capture and signal readout [13]. One such electrochemical technique is electrochemical impedance spectroscopy (EIS). With EIS, the impedance of the electrode–electrolyte interface is studied across a range of frequencies to establish information regarding the interface, electron-transfer and diffusional behaviour. Changes in impedance can be indicative of DNA detection. Differential pulse voltammetry (DPV) is another electrochemical technique of interest and can be used to sensitively investigate electron transfer to and from an electrode surface [14]. Previously, DNA sensors exploiting EIS and DPV have been produced for the detection of sepsis biomarker interleukin-6 (IL-6) [15], antibiotic resistance [16] and fusion genes [17]. 

Open-circuit potentiometry (OCP) is an electrochemical technique which is used to measure the potential of an electrochemical system when there is no current flowing. It is a simple and highly sensitive technique and is useful for determining the potential within a system, particularly when a DNA sequence of interest is introduced. Previously, OCP has been exploited for the development of a platinum nanoparticle-based immunoassay for the detection of human chorionic gonadotropin hormone [18].

Field-effect transistors (FETs) can be used as a highly sensitive and low-cost approach for the detection of ctDNA as they can act as signal amplifiers [19]. FETs have the ability to achieve high sensitivity through amplification of the biorecognition signal resulting from complementary ctDNA from a clinical sample binding to probe DNA immobilised either at the FET gate or across the semiconductor channel. Such an approach could eliminate the need for polymerase chain reaction (PCR)-type amplification of tumour DNA mutations for detection [20], which is particularly attractive for use at the PoC as PCR both increases time to result and has high associated laboratory costs [20]. FETs typically consist of three terminals: the gate, source and drain. Controlling the voltage applied to the gate and drain terminals influences the current flow between the source and drain. As well as providing amplification, FETs offer the ability to directly transduce a change in voltage or current, useful for data capture and readout. This method is simpler than electrochemical techniques such as EIS, which requires circuit fitting to an equivalent circuit to interpret results, and is ultimately more amenable to a PoC setting. FETs, and particularly organic semiconductor variants which feature inherent advantages such as low-cost production and compatibility with flexible substrates [21,22], can also be mass produced via processes such as vacuum thermal evaporation [23,24] and printing [25], which results in high-volume/low-cost production.

In this paper, we describe the steps taken to develop a FET-based sensor for the detection of common tumour protein mutation TP53 in ctDNA, particularly with the goal in mind of developing a route towards a liquid biopsy. We use an electrochemical-based approach initially, with a particular focus on the measurement technique OCP as the most representative technique by which a FET would read changes in solution, to optimise the electrode sensing pad for DNA hybridisation and detection, and then couple the sensing pad to the FET gate for signal transduction and DC voltage readout. The optimisation steps described herein are essential to be able to create a highly sensitive and specific sensor for a common TP53 mutation which is suitable for interfacing with a field-effect transistor sensor.

## 2. Materials and Methods

### 2.1. Methodology

Polycrystalline gold electrodes (PGEs) featuring a diameter of 2 mm were purchased from IJ Cambria (Llanelli, UK). Gold microelectrodes with diameters ranging from 1000 to 100 µm were fabricated in-house at the Strathclyde Institute of Photonics cleanroom facility. Commercially available n-channel enhancement mode field-effect transistors (2N7000) (FETs) were obtained from Farnell (Leeds, UK) and featured three terminals: gate, source and drain. Gold (Au) wire for the extended FET gate was purchased from Agar Scientific Ltd (Stansted, UK). All solutions were prepared using deionised (DI) water (Sigma Aldrich, Dorset, UK). Ferri-ferrocyanide (FF-C) (Fe[CN]_6_^3−^ + Fe[CN]_6_^4−^) solution was prepared from the constituent components ferricyanide and ferrocyanide, which were obtained from Arcos Organics (Thermo Fisher Scientific, Geel, Belgium). All other chemicals including 3-Mercapto-1-propanol (MCP) and tris(2-carboxyethyl) phosphine (TCEP) were purchased from Sigma Aldrich. Table 1 provides information regarding the DNA sequences used for the study, including the DNA probe with and without spacer [SP18] and the complementary target tested. ctDNA mutation TP53 was chosen for investigation. All oligos were obtained from Sigma Aldrich and prepared in TRIS buffer. The complementary probe and target sequences were each 21 base pairs long. A 53-mer oligo was used as the noncomplementary control target. The same probe was used when testing noncomplementarity (specificity); only the target was substituted in this case. Figure S1 and Table S1 of the supporting information include additional data showing the specificity of the electrochemical part of the system to another ctDNA sequence (KRAS G12D) with only a single-base mismatch between the mutant and wildtype sequences.

Prior to functionalisation of the Au electrode ‘sensing pad’, all electrodes were cleaned following a well-established protocol involving immersion in Piranha solution, polishing with alumina slurry, sonicating for 5 min in DI water and then electrochemically cleaning by cyclic voltammetry (CV) in 0.1 M sulphuric acid (H_2_SO_4_) to completely regenerate the PGE surface. The cleaning procedure produces a smooth and homogenous electrode surface for subsequent DNA functionalisation. Surface functionalisation was performed through the creation of a SAM (self-assembled monolayer) on the electrode surface, which was found to be reproducible between experiments. Electrodes were nominally incubated with 1 µM probe DNA and 5 µM tris(2-carboxyethyl) phosphine (TCEP) in DI water overnight. Electrodes were then rinsed in DI water to remove unbound DNA. Next, the electrodes were backfilled using 1 mM mercaptopropanol (MCP) (95% purity) and 5 mM TCEP in DI water for 1 h and then rinsed thoroughly in DI water.

### 2.2. Characterisation

**Figure 1:**
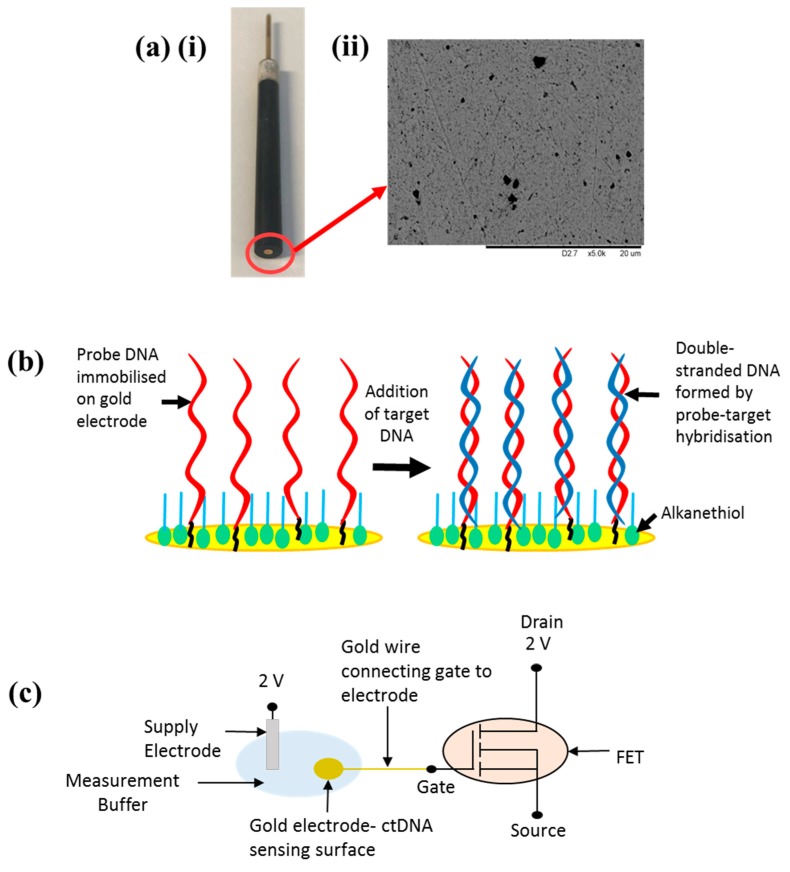
(**a**) (i) Polycrystalline gold electrode (PGE) acting as ‘sensing pad’ element of (field-effect transistor) (FET) sensor device. (ii) Scanning electron microscope (SEM) image of Au PGE electrode surface at × 5 k magnification. (**b**) Schematic of the sensing pad surface showing probe DNA with and without the hybridised complementary DNA target. (**c**) FET sensor equivalent circuit showing Au electrode sensing pad connected via an extended gate to the FET input side.

Electrochemical measurements on the PGE ‘sensing pad’ were performed using a three-electrode cell. In addition to the PGE, the cell was completed by using a platinum wire counter electrode and a saturated Ag/AgCl reference electrode. All measurements were carried out using a potentiostat (PalmSens PS4, PalmSens, Houten, Netherlands). Once cleaned, electrodes were electrochemically characterised in a measurement solution of 2 mM FF-C. EIS, DPV and OCP measurements were performed consecutively in the same measurement solution to characterise the PGE. EIS measurements were performed between 100 kHz and 0.1 Hz at open-circuit potential using an amplitude of 10 mV r.m.s, and the complex impedance spectra were fitted to a Randles equivalent circuit to determine the charge transfer resistance (R_CT_). DPV was performed between –0.2 and 0.5 V and the peak current (I_pk_) was extracted. OCP was performed for 10 s for regular static characterisation measurements, and up to 7200 s (2 h) for continuous measurements. Scanning electron microscope (SEM) (TM-1000, Hitachi, Tokyo, Japan) images were taken of the Au (PGE) electrode surface to gain an impression of the surface profile. SEM images were performed by scanning a 30 × 30 µm area, at a magnification of ×5.0 k. The FET setup utilised a source-follower configuration (Figure 1c), with the Au electrode sensing pad connected to the FET gate via Au wire in the form of an extended gate. The drain and gate terminals were powered at 2 V DC (V_GS_ = V_DS_ = 2 V) and the source was grounded. The average voltage (potential) at the FET output across the source and drain was measured as a function of DNA concentration and compared to electrode sensing pad OCP measurements. FET potential measurements were recorded using a highly sensitive impedance analyser (Metrix MX 57EX). For every continuous OCP measurement and FET measurement performed, a minimum of three experiments were performed which were found to be entirely internally self-consistent. This negligible intra-experimental variation gave confidence that the reported effect of OCP as a function of target concentration and relative differences between complementary and noncomplementary sequences were reproducible. Representative data is displayed and error bars have not been included in this instance as a result of inter-experimental variation being higher, thus making error bars unrepresentative of the true effect. Inter-experimental variation was high because of electrode-to-electrode variation and variations in the electrode history, in addition to electrode cleaning processes. Furthermore, variations in the FET behaviour were observed, and going forward, being able to microfabricate FET devices could help to eradicate any batch-to-batch variations in FET response. In summary, these various factors related to both the electrode and FET structures will be the subject of future standardisation experiments.

## 3. Results and Discussion

### Sensor Setup

To effectively establish the optimum measurement setup for the FET sensor for TP53 detection, various experiments were performed to optimise the ‘sensing’ side of the device (i.e., the gold electrode). PGEs were chosen for this purpose and despite their relatively high cost compared to screen-printed electrodes (SPEs), they offer a higher quality Au surface which can be polished to a mirror finish, resulting in improved DNA detection, sensitivity and reproducibility between measurements [16]. Previously, PGEs have found use as enzymatic biosensors for peroxide detection and as chemical sensors amongst others [26,27]. For the investigation of electrode diameter, microfabricated gold electrodes were used. These were fabricated in-house in a cleanroom via a series of photolithography and etching steps. The 1000 µm diameter electrode was treated as the most comparable in terms of dimensions to the PGEs (2 mm) and would be classed as macroelectrodes. Smaller electrodes were treated as significantly different in diameter and for the purposes of this study were referred to as microelectrodes. A three-electrode cell was developed for electrochemical measurements featuring the PGE working electrode (WE), platinum counter electrode (CE) and Ag/AgCl reference electrode (RE). The PGE used for electrochemical measurements is presented in Figure 1a (i), with the Au electrode surface where the reaction takes place circled in red for clarity. To investigate the quality of the Au electrode surface, SEM was performed on the bare electrode. The SEM image shown in Figure 1a (ii) shows that the PGE features a predominantly smooth surface profile, with only a few voids across the surface featuring nonhomogenous particle sizes.

The sensor detection mechanism relies upon electrochemical changes at the Au WE surface when target DNA is attached to the surface, ultimately resulting in a change of the open-circuit potential which can be measured as a voltage change, suitable for readout via a FET. Figure 1b depicts a schematic of the DNA attachment protocol on the Au sensing pad surface, whereby initially single-stranded (s-s) probe DNA is immobilised through semi-covalent bonding of the alkanethiol group to the gold, followed by the addition of target DNA of various concentrations. Probe–target hybridisation enables the formation of double-stranded (d-s) DNA. More information regarding the attachment process can be found in related work by Keighley et al. [28]. Electrochemical measurements were used to monitor changes between the probe-only surface (pre-target) and the surface with target DNA added (post-target) to ascertain the effect of target hybridisation on the electrode, and optimise various electrode parameters to obtain the greatest change between pre-target and post-target measurements. Upon optimisation of the electrode sensing pad, the FET element of the sensor was introduced. The FET sensor comprises a commercially available transistor which acts as the transducer. The FET gate is connected in series to the electrode sensing pad via Au wire, therefore the FET threshold voltage (V_t_) should remain approximately constant. The system comprises a supply electrode, ultimately controlling the voltage applied to the gate (V_GATE_), the sensing pad electrode which behaves like a floating gate (V_PAD_) where DNA hybridisation occurs and the FET itself acting as transducer. It is possible to model the voltage dropped at the gate electrode through the addition of the sensing pad via an extended gate arrangement by relating the voltage to both the capacitance of the gate (C_GATE_) and the capacitance of the semiconductor channel (C_FET_) and their respective areas [29,30]. According to an investigation performed in [29] by S. P White et al., in order to reduce the effective voltage drop, the area of the gate must be at least 150 times greater than the area of the semiconductor. If this ratio is not satisfied, and the area of the gate is lowered compared to the semiconductor area, then transistor turn-on is not as sharp, FET ON current is reduced and there is also a noticeable hysteresis. In addition, using a simple lumped capacitor model as outlined in [29] where it is assumed that the semiconductor is held at ground and there is zero gate current, if C_GATE _>> C_FET_, then V_PAD_ ≈ V_GATE_, a critical feature to ensure that the sharpest transistor turn-on is achieved since negligible voltage is dropped. For the system presented here, the various capacitances and areas are unknown since a commercially available FET was used as a proof of concept; however, future device design would incorporate these crucial area and capacitance ratio elements to ensure as little voltage is dropped as possible, maintaining low-voltage device operation and ultimately increasing the sensitivity of the proposed system. However, in the case of the system presented, since the FET is connected in a source-follower configuration, we can make an approximation and model the device in the ideal situation, whereby the potential change induced by the addition of target DNA (ΔV_DNA_) should be the only system variable. Therefore, the expected system output should be approximately equal to the potential change through DNA addition as detailed in Equation (1):

∆V_OUT_ ≈ ∆V_DNA_ (1)

The advantage of using the extended gate approach has been demonstrated previously [22,31]; however, the main reason for doing so is to avoid additional complexity resulting from device encapsulation, as the transducing element (FET) is isolated from the sensing side. Figure 1c shows a schematic representation of the sensor setup, with the Au electrode sensing pad immersed in measurement solution, attached in series to the FET gate via Au wire.

**Figure 2:**
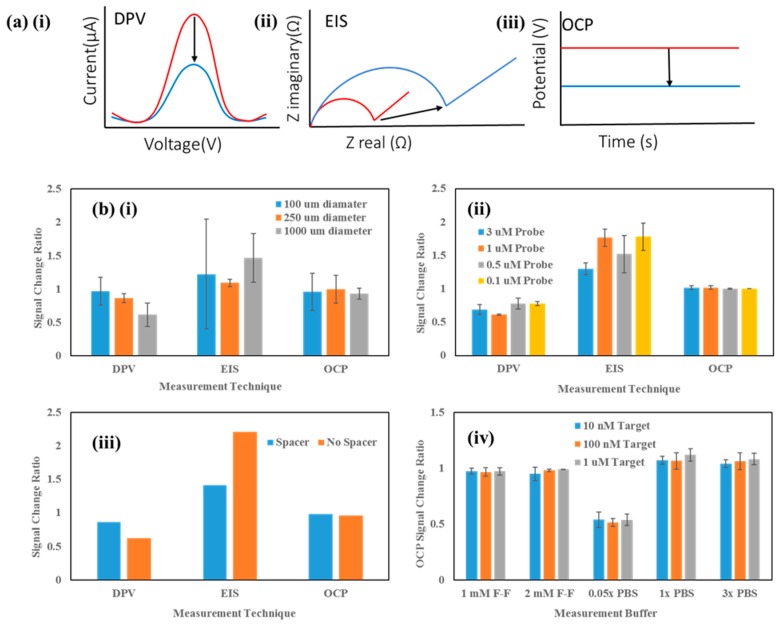
(**a**) Example electrochemical measurements (Differential pulse voltammetry (DPV) (i), Electrochemical impedance spectroscopy (EIS) (ii) and Open-circuit potentiometry (OCP) (iii)) depicting the effect of hybridisation with complementary target DNA compared to the initial ‘pre-target’ measurements. Pre-target data is shown in red and post-target data in blue. (**b**) Au electrode sensing pad optimisation experiments: (i) electrode diameter, (ii) DNA probe concentration, (iii) DNA probe with/without spacer and (iv) effect of measurement buffer on different concentrations of target DNA.

Figure 2a shows representative graphs of what should occur when target DNA is hybridised to the probe DNA on the electrode surface with regards to electrochemical measurements differential pulse voltammetry (DPV) (i), electrochemical impedance spectroscopy (EIS) (ii) and open-circuit potentiometry (OCP) (iii). DPV is a highly sensitive voltammetric method and features a series of voltage pulses superimposed on a potential linear sweep (e.g., staircase effect). The current is measured prior to each voltage change. EIS is used to monitor complex impedance changes within a system by applying a small AC voltage pulse and measuring the real and imaginary parts of the impedance as a function of frequency. OCP refers to the potential of an electrochemical system where no current is flowing and is the most accurate representation from an electrochemical perspective of what happens to the potential change within a FET system when detecting DNA. Upon attachment of target DNA, the electrode surface is deemed to be more ‘blocked’ (physically and electrostatically), therefore hindering the ability of an electrochemical redox mediator such as FF-C to penetrate to the electrode surface. This manifests itself as an increase in the charge transfer resistance (*R_CT_*) portion of EIS, and with an increased resistance, the DPV peak current and open-circuit potential should theoretically decrease according to Ohm’s law (see Figure 2a).

Before connection to the FET side of the sensing circuit, the Au PGE sensing pad was optimised electrochemically via DPV, EIS and OCP measurements. Various parameters were investigated to optimise sensitivity of the Au pad for DNA detection including the electrode size, probe DNA concentration, probe DNA with and without a spacer between the DNA sequence and the thiol group and the composition of the measurement buffer in which measurements were performed.

Firstly, the effect of electrode size on the DNA measurement was investigated. Figure 2b (i) shows the effect of electrode diameter (100, 250 and 1000 µm) on the signal change between pre-target and post-target electrochemical measurements. These experiments were performed on microfabricated electrodes to obtain the smaller electrode diameters for comparison. As expected, DPV and OCP post-target resulted in a % decrease, whereas EIS resulted in an increase in *R_CT_* after target addition. It is clear that the greatest signal change regardless of measurement technique is produced by the largest (1000 µm) diameter electrode, and it also provides the most consistent change for OCP measurements. Therefore, coupled with the more reproducible surface and the trend of greater signal change with larger electrode diameter, the 2 mm PGE was chosen as the optimum electrode to take forward into further optimisation.

Next, the concentration of probe DNA in immobilisation buffer was investigated to optimise the probe DNA density. Figure 2b (ii) shows DPV, EIS and OCP data for four different concentrations of probe DNA in the immobilisation solution (0.1, 0.5, 1 and 3 µM). Whilst the OCP appeared to show little difference, DPV and EIS showed the greatest decrease and increase, respectively, post-target addition for the 1 µM probe. To finalise the probe optimisation, a probe was designed which featured a spacer (SP18) at the 5’ end to ascertain whether extending the recognition sequence away from the surface and further into solution resulted in improved hybridisation of the target DNA, and ultimately, a greater change in OCP. For all measurements performed, the effect of the spacer was profound (Figure 2b (iii)) and actually resulted in a smaller change post-target than the original probe without spacer. This was an interesting find since in the literature, for electrochemical assays, there are many reports of spacers actually improving sensitivity [32,33] and therefore it was deemed worthwhile to investigate the effect of a spacer for this system. However, the fact that introduction of a spacer in this case resulted in a smaller change post-target could be an advantage for a FET-based system where the Debye length is a critical parameter to take into account. Therefore, going forward, a 2 mm diameter PGE electrode was used for all experiments, featuring a probe concentration of 1 µM with no spacer. The final optimisation experiment performed was to investigate different measurement buffers to optimise the signal change with regards to OCP. Figure 2b (iv) shows the OCP signal change ratio using three different target DNA concentrations for comparison (10 nM, 100 nM and 1 µM). Five measurement buffers were tested (1 mM FF-C in 1× PBS, 2 mM FF-C in 1× PBS, 0.05× PBS only, 1× PBS only and 3× PBS only) (PBS = phosphate-buffered saline, pH ~ 7.2). Whilst FF-C is a common electrochemical redox mediator which will fix a well-defined OCP, the two ions involved have 4^−^ and 3^−^ charges giving rise to a high ionic strength, and therefore PBS was investigated to gauge the effect purely of salt concentration on the OCP of the system, particularly important for subsequent FET measurements where the Debye length is an important consideration to avoid potential electrostatic screening effects resulting in significantly reduced sensitivity of the FET system for DNA detection [34]. From Figure 2b (iv), it is clear that the 0.05× PBS measurement buffer provided the greatest signal change ratio (OCP reduction) of all the buffers. In all cases, there was clear differentiation between target concentration and OCP, which is promising for sensitive TP53 detection at concentrations in the nM range. Based on these results, the best measurement buffer for subsequent measurements was chosen as the 0.05× PBS.

**Figure 3:**
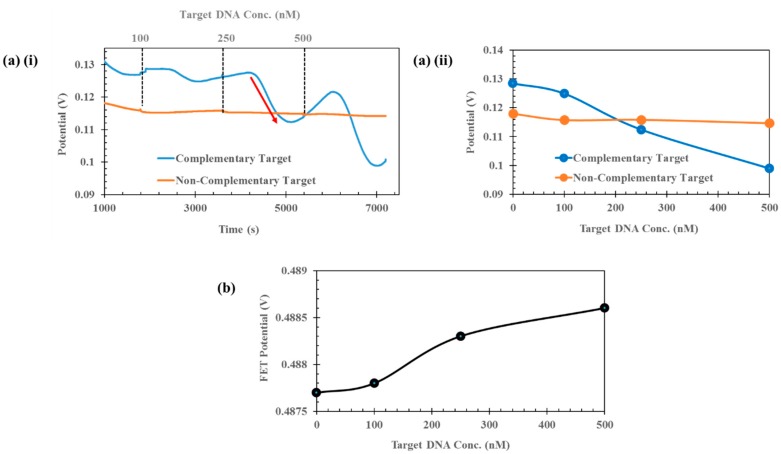
(**a**) (i) Continuous OCP measurement measuring change in potential as a function of increasing target DNA concentration over time. Graph shows the effect of complementary and noncomplementary target sequences on OCP. (**a**) (ii) Average OCP potential as a function of increasing target DNA concentration for complementary and noncomplementary DNA target sequences. (**b**) Average FET potential as a function of increasing target DNA concentration for complementary DNA target.

Primed with optimised probe data and the optimum measurement buffer for OCP measurements, the next step involved investigating how the open-circuit potential of the sensing system varied over an extended period of time. From Figure 2b (iv), it is clear that OCP varied as a function of target concentration, and the observed decrease was greater as target concentration increased. However, these OCP measurements are based on 10 s long measurements of OCP, and do not take into account longer-term changes in system stability. Figure 3a (i) shows the effect of performing a ‘continuous’ OCP measurement over a period of 7200 s (2 h) to investigate the sensitivity of the Au PGE sensing pad to changes in target TP53 concentration. (Data from 0 to 1000 s is not shown due to the solution equilibrating and the data featured some noise which resulted in a poor representation of the subsequent potential changes). After a period of 30 min to allow the measurement buffer to stabilise (mixing or using a stirring bar could reduce the time required), the first concentration of target DNA (100 nM) was added to the mix. Every 30 min, an increased concentration of target DNA (250 nM followed by 500 nM) was added to establish system sensitivity around physiologically relevant levels [33]. It is clear that for the case of the complementary probe and target DNA sequences, as the concentration of target DNA was increased, there was a larger decrease in OCP. The red arrow in Figure 3a (i) highlights the effect of adding target DNA to the solution, whereby OCP decreased temporarily to a new steady-state position at that point in time. For comparison, a noncomplementary target sequence was examined using identical conditions to investigate the specificity of the developed sensor. It is encouraging to note that for the noncomplementary case, there was no tangible change in OCP when target DNA between 100 nM and 500 nM was added to the system, indicating that no hybridisation was occurring between the probe and target. Figure 3a (ii) shows the new steady-state potential of the system when target DNA has been added as a function of target DNA concentration. In the case of the complementary target, the OCP potential decrease between the pre-target situation and after application of 500 nM target DNA was 23.01%, whereas for the noncomplementary sequence, the change over the same concentration range was only 2.78%. A useful biosensor should not only be sensitive, but specific also to the particular parameter of interest (ctDNA) which this system clearly offers.

Since the OCP was proven to be sensitive to changes in target DNA, the final stage was to evaluate the low-cost FET as a means to monitor changes in potential, avoiding the need for more complex and costly OCP measurements. The sensor setup shown in Figure 1c was used, whereby the Au PGE sensing pad was connected in series to the FET gate via Au wire with a gate voltage and drain voltage of 2 V. An identical experiment to that shown in Figure 3a was performed using a complementary target sequence. Figure 3b shows the average FET potential upon addition of target DNA as a function of DNA concentration. It is clear that as the concentration of target DNA was increased from the pre-target condition up to a maximum of 500 nM, the FET potential increased from 487.7 mV to 488.6 mV, an increase of ~ 1 mV. Whilst this change is small, it is indeed measurable. Possible methods to increase the FET potential change have been hypothesised, including the use of thin-film transistors such as organic FETs or organic thin-film transistors (OTFTs) [22,23,24]. Such devices feature significantly smaller feature sizes with channel lengths in the micron range enabling greater charge carrier mobility and hence potential change. A second method to improve sensitivity of the sensor would be to take measurements using a semiconductor parameter analyser, enabling the sourcing and measurement of small voltages and currents (femto amps range), and the FET could act as an amplifier and current changes could be measured instead of potential. Optimising the applied gate and drain voltages to the FET could also improve sensitivity, and biasing the device in the subthreshold regime could be a good starting point for sensitivity improvement. However, the current setup does provide a modest change in potential for small changes in target DNA concentration, eliminating the need for more complex and costly electrochemical detection techniques. Interestingly, whilst the OCP decreases with increasing concentration of target DNA, the FET potential increases. This is most likely a result of how the FET is biased, and a change in biasing voltages or use of a p-channel FET could result in a potential decrease. However, regardless of the increase/decrease, the important aspect is that there is an apparent potential change for DNA target concentrations in the nM range and it is consistent.

With regards to sensor performance, a similar study using a FET-based approach looking at PNA (peptide nucleic acid)–DNA detection was performed [31]. In this case, they found a potential shift of ~70 mV when using 1 µM DNA. This is highly comparable with the change in OCP we have observed in Figure 3a, despite our lower DNA target concentration. In addition, a sensor using a GaN nanowire extended-gate FET was developed previously for TP53 detection [35]. The approach enabled very high sensitivity (attomolar range) and was specific between complementary and noncomplementary sequences. Another system using a FET biosensor for detection of mutant TP53 made use of an n-type CMOS MOSFET as the transducer [34]. The system compared both wildtype and mutant TP53 DNA, and after binding, a significant increase in FET drain current was noted with the wildtype sequence (~250 µA), whereas for the mutant sequence, the drain current only increased by ~20 µA for the same 100 nM target DNA concentration, confirming specificity of the system. In [36], the authors also provide validation for our increase in FET potential, since the TP53 protein which is positively charged shields the consensus DNA on the gate surface, and an increase in positive charge on the gate surface will increase the FET channel conductance, and thus the drain current, and in our case, potential, will increase. Whilst highly specific to TP53 DNA, the main drawback of these systems is their fabrication complexity as well as relatively high cost for development.

This study provides a clear path towards using a FET-based biosensor for rapid (<30 min) and sensitive (nM) detection of TP53 as a promising route towards establishing point-of-care measurements relevant to cancer diagnosis. Next steps will involve optimising the FET sensitivity and initiating steps to microfabricate FETs to benefit from lower voltage operation, enhanced sensitivity and even device integration on flexible substrates more suitable for use at the point of care.

## 4. Conclusions

A low-cost, sensitive and specific electronics-based sensor system has been developed for the detection of TP53 mutations, as a route towards a simple, noninvasive assay for tumour formation. The proposed sensor features a field-effect transistor transducer for signal amplification and voltage readout. The sensor features a polycrystalline gold electrode as the sensing pad for DNA hybridisation, connected in series using an extended gate setup to the transistor gate for transduction. The sensor has been optimised with particular attention to the sensing side, and aspects including electrode diameter, probe DNA concentration, probe DNA sequence and measurement buffer have been explored electrochemically to ascertain the best parameters for detection. Electrochemical techniques including OCP have been explored as a model for subsequent potential changes through the FET system, for TP53 detection at different target concentrations. The sensor is able to sensitively respond via OCP to target concentrations as low as 100 nM within minutes, and is selective when a noncomplementary DNA target strand is introduced. When the sensing pad is connected to a low-cost, commercially available field-effect transistor, the addition of target DNA up to a concentration of 500 nM resulted in a FET potential change of ~1 mV, which could be improved upon through the use of microfabricated FET devices or using changes in FET output current. The system paves the way towards a low-cost, sensitive and selective field-effect transistor biosensor for the accurate and rapid detection of circulating DNA biomarkers indicative of tumour formation without the requirement for more complex and costly electrochemical methods of detection.

## Figures and Tables

**Table 1:** DNA Probe and Target Sequences.

Sequence Name	Modification	Sequence 5’–3’
Mutant type (MT) P53 probe with spacer	5’ thiol-CH SP18	TTTGAGGTGCATGTTTGTGCC
Mutant type (MT) P53 probe without spacer	5’ thiol-CH	TTTGAGGTGCATGTTTGTGCC
Mutant Type (MT) P53 target	N/A	GGCACAAACATGCACCTCAAA
Noncomplementary DNA target	N/A	GGGAGAGAGAACTGGACGATGATGGGAATGGACTAAGGATGACGGAAACAGAT

## Supplementary Materials

The following are available online at https://www.mdpi.com/2079-6374/9/4/141/s1, Figure S1: Voltammetric peak current percentage signal change for mutant and wildtype KRAS G12D target sequences; Table S1: KRAS G12D mutation oligo sequences.
