# Supplementary material for: Establishing a Field-Effect Transistor Sensor for the Detection of Mutations in the Tumour Protein 53 Gene (TP53)—An Electrochemical Optimisation Approach
Source: Biosensors (Basel). 2019 Dec 6;9(4):141. doi: 10.3390/bios9040141 (PMC6956290; doi:10.3390/bios9040141)
Supplement: Supplementary file 1 [file biosensors-09-00141-s001.pdf]

Supporting Information

Establishing a Field-Effect Transistor Sensor for the Detection of Mutations in the Tumour Protein 53 Gene (TP53) – An Electrochemical Optimisation Approach

Lisa Crossley [1], Bukola Attoye [1], Vincent Vezza [1], Ewen Blair [1], Damion K. Corrigan [1], and Stuart Hannah [1,*]

[1] Department of Biomedical Engineering, University of Strathclyde, 40 George Street, Glasgow, G1 1QE; lisa.crossley.2015@uni.strath.ac.uk (L.C); bukola.omolaiye@strath.ac.uk (B.A); vincent.vezza@strath.ac.uk (V.V); ewen.blair@strath.ac.uk (E.B); damion.corrigan@strath.ac.uk (D.C); stuart.hannah@strath.ac.uk (S.H)
* Correspondence: stuart.hannah@strath.ac.uk

Figure S1 presents the effectiveness of the electrochemical system developed for optimisation of the FET system shown in the manuscript for discriminating the presence of a single-base mismatched oligo for a prominent ctDNA mutation, KRAS G12D, with regards to the change in voltammetric peak current after target hybridisation. This figure has been included to represent the ability of the electrochemical system used in this study for FET optimisation to detect a single-base mismatch to prove specificity of the system. The figure shows data related to KRAS G12D, however, we believe our sensor would respond equally well to single-base mismatch P53 mutations.

Table S1 contains the sequences used for the KRAS G12D study shown in Figure S1 including the mutant DNA probe sequence and the mutant and wildtype DNA target sequences for the oligo sequences tested. Much longer PCR products of these targets were also tested for comparison. Figure S1 shows a clear discrimination between the mutant and wildtype sequence for both the standard oligo and PCR

products tested. The mutant target results in a negative percentage change in each case, whereas the wildtype shows a slight DPV percentage increase. It is clear that the mutant PCR product results in a significantly greater (-316 %) percentage change compared to the oligo (-17 %) which is to be expected based on the length of sequences involved.

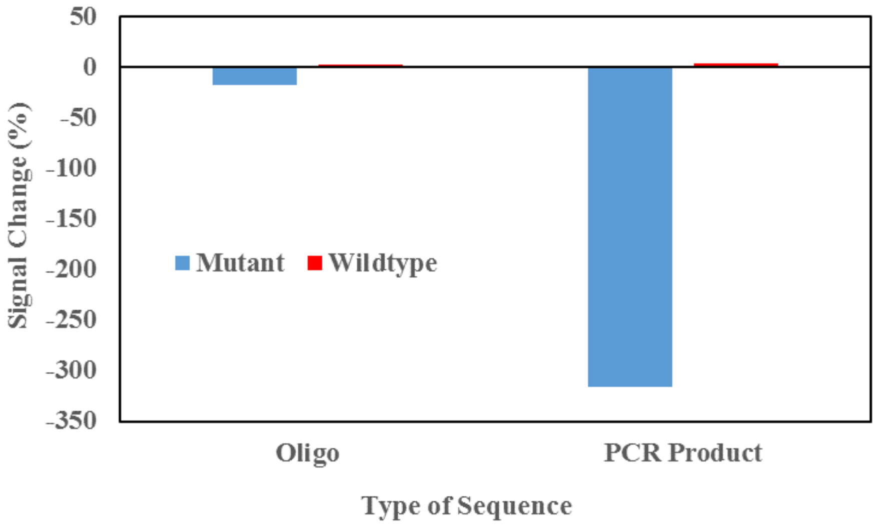

Figure S1. Voltammetric peak current percentage signal change for mutant and wildtype KRAS G12D target sequences. Standard oligo targets and PCR products are included.

Table S1. KRAS G12D mutation oligo sequences. Mutations are highlighted in bold.

Sequence Name	Sequence 5′ – 3′
Mutant Type (MT) KRAS G12D probe	AGTTGGAGCTG**A**TGGCGTAGGCA
Mutant Type (MT) KRAS G12D target	TGCCTACGCCA**T**CAGCTCCAACT
Wild type (WT) KRAS G12D target	TGCCTACGCCA**G**CAGCTCCAACT
